# Case Report: unmasking pseudo-homozygosity in *CYP21A2*: intergenerational gene conversion expansion and the necessity of multimodal genetic testing

**DOI:** 10.3389/fendo.2026.1884514

**Published:** 2026-08-18

**Authors:** Zuhal Altintas, Sefanur Karaca, Ayhan Coskun

**Affiliations:** 1 Department of Medical Genetics, Faculty of Medicine, Mersin University, Mersin, Türkiye; 2 Department of Obstetrics and Gynecology, Division of Perinatology, Mersin University Faculty of Medicine, Mersin, Türkiye

**Keywords:** *CYP21A2*, gene conversion, intergenerational expansion, MLPA, prenatal diagnosis, pseudo-homozygosity

## Abstract

*CYP21A2* gene mutations causing Congenital Adrenal Hyperplasia (CAH) exhibit high allelic complexity. Rare meiotic events, such as *de novo* expansion of deletion boundaries, can obscure true genotypes, leading to ‘pseudo-homozygosity’ and diagnostic pitfalls. This study characterizes a rare intergenerational expansion of a maternal *CYP21A2* gene conversion and demonstrates the critical role of multimodal genetic testing in resolving complex inheritance paradoxes during prenatal counseling. A family with classical salt-wasting CAH, involving parents who are second cousins, was investigated. Initial screening suggested p.Ile173Asn homozygosity in their third child (designated as V-3 in the pedigree chart), who exhibited severe birth virilization and was initially assigned male but later reassigned female. In-house Multiplex Ligation-dependent Probe Amplification (MLPA) and Sanger sequencing using two independent unique primer sets were integrated to assess copy number variations and resolve parental discrepancies. Maternal probe analysis revealed a heterozygous Exon 1–3 gene conversion, leaving the p.Ile173Asn locus intact on her wild-type allele. However, this conversion meiotically expanded to Exons 1–4 in the affected child (V-3). This expansion physically eliminated the maternal wild-type primer template, causing PCR allele dropout and a false pseudo-homozygous Sanger pattern, which biologically corresponds to true functional hemizygosity *in vivo*. The father’s suspected deletion was ruled out as probe-binding interference from his heterozygous p.Ile173Asn mutation. Guided by these resolved genotypes, early prenatal dexamethasone management and subsequent twelfth-week Chorionic Villus Sampling successfully confirmed a normal wild-type female fetus (46,XX), resulting in a healthy term delivery. In conclusion, the *CYP21A2* locus is highly dynamic, and integrating dosage-sensitive assays with unique primers is mandatory for accurate genetic counseling and successful prenatal management in high-risk pregnancies.

## 1 Introduction

Congenital Adrenal Hyperplasia (CAH) is a group of autosomal recessive disorders, with approximately 95% of cases resulting from mutations in the *CYP21A2* gene (1). The molecular landscape of the *CYP21A2* locus, situated within the highly complex RCCX modular complex (comprising the *RP2*, *C4B*, *CYP21A2*, and *TNXB* genes) on chromosome 6p21.3, is among the most challenging regions for genetic analysis (2, 3). The high sequence homology between the functional *CYP21A2* gene and its truncated, non-functional pseudogene, *CYP21A1P*, facilitates frequent intergenic recombination events. Quantitatively, small gene conversions that transfer variants from *CYP21A1P* account for approximately 70% of affected alleles, whereas large 30-kb deletions or unequal crossovers that form *CYP21A1P–CYP21A2* chimeric genes account for 25–30% of cases (4, 5). Spontaneous *de novo* point mutations or intragenic insertions/deletions (indels) constitute only about 5% of the pathogenic variant spectrum (6). This architectural complexity frequently yields compound mutations and complex genotypes, necessitating advanced diagnostics.

While the majority of pathogenic alleles are derived from micro-conversions or stable genomic deletions, the repetitive architecture of the RCCX region acts as a substrate for further chromosomal instability (7, 8). This instability can lead to dynamic, intergenerational changes in mutation boundaries during meiotic transmission, a phenomenon that is rarely captured but has profound clinical and prenatal implications (7, 9).

A critical diagnostic pitfall in the molecular management of CAH is “pseudo-homozygosity”. This phenomenon describes a clinical scenario where an individual appears to be homozygous for a specific mutation on standard sequencing but is, in fact, hemizygous. This occurs when a large structural rearrangement or an expanded gene conversion tract on one allele physically removes or prevents the amplification of the wild-type template. Consequently, during polymerase chain reaction (PCR), only the mutant allele from the other parent is captured, a process known as allele dropout, leading to an erroneous homozygous interpretation (10, 11). Furthermore, point mutations located within Multiplex Ligation-dependent Probe Amplification (MLPA) probe hybridization sites can lead to false-positive deletion signals due to probe-binding interference, further complicating the interpretation of copy number variations (CNVs). Such misinterpretations can lead to incorrect genotype-phenotype correlations and catastrophic errors in genetic counseling and sex assignment at birth (12, 13).

Recent advancements in genomic techniques, including MLPA and long-read sequencing, have revolutionized the detection of these complex rearrangements (14, 15). However, when advanced sequencing platforms are unavailable or external genetic reports yield Mendelian discrepancies, the tactical integration of dosage-sensitive assays with highly specific, independent primer designs remains a powerful, accessible, and mandatory tool for validating physical deletion boundaries (16).

In this study, we characterize a rare case of intergenerational meiotic expansion of a maternal *CYP21A2* gene conversion tract, which shifted from an Exon 1–3 boundary in the asymptomatic mother to an Exon 1–4 boundary in her affected daughter (designated as V-3 in the pedigree chart), born to consanguineous parents who are second cousins. This expansion demonstrates the highly dynamic nature of gene conversion tracts during meiotic recombination. By integrating MLPA and Sanger sequencing using two independent sets of unique (*uniq*) primers, we demonstrate how multimodal genetic testing can resolve pseudo-homozygosity. Furthermore, we highlight the clinical impact of these findings on resolving neonatal sex assignment discrepancies, ensuring precise genetic counseling, and executing successful prenatal management in a subsequent high-risk pregnancy.

## 2 Case presentation

### 2.1 Clinical background and family history

A 38-year-old female (Gravida 4, Para 3, Living 3) presented to the Perinatology clinic for comprehensive preconceptional counseling and prospective prenatal management. Her obstetric history was notable for normal spontaneous vaginal deliveries (NSVD) in her first three pregnancies, resulting in two healthy older daughters (V-1 and V-2) and one affected child. Her family history was highly significant for classical salt-wasting CAH on the maternal side, with two prior maternal cousins clinically managed for severe adrenal crises. The parents shared a known consanguineous background, specifically being second cousins (sixth-degree blood relatives whose biological fathers were first cousins born to maternal sisters), which perfectly aligned with the consanguinity mapped in the updated pedigree chart.

Their third child (designated as V-3 in the pedigree chart and currently followed up as a 12-year-old girl) had a complex medical and surgical history. Born with ambiguous genitalia exhibiting severe virilization (Prader Stage IV–V, blood pressure 82/45 mmHg, marked hyponatremia of 121 mEq/L, and hyperkalemia of 6.8 mEq/L), the neonate was initially assigned male. Physical examination subsequently revealed an external female phenotype but with marked virilization, for which clitoroplasty and vaginoplasty had been previously performed at an external center. On March 13, 2015, an official multidisciplinary Gender Assignment Committee formally designated the child’s sex assignment toward the female gender. Pelvic ultrasonography (USG) confirmed the absence of gonads within the scrotal folds; instead, an intact uterine structure and bilateral gonads were identified within the abdominal cavity. Based on these comprehensive clinical, imaging, and cytogenetic findings (46,XX), a definitive diagnosis of CAH due to 21-hydroxylase deficiency was established. Following extensive counseling with the family regarding the high potential for future fertility, the therapeutic and surgical management was definitively aligned toward female gender differentiation. Following these comprehensive endocrine and multidisciplinary evaluations, the child was initiated on standard hormone replacement therapy (titrated maintenance doses of hydrocortisone at 12 mg/m²/day and fludrocortisone at 0.1 mg/day).

Prior to the patient’s referral to our center, external genetic screening for this affected child and the parents had yielded a significant inheritance paradox:

- Affected Child (V-3): Reported as homozygous for the p.Ile173Asn (c.518T>A) mutation via Sanger sequencing.
- Father: Reported as a heterozygous carrier for the p.Ile173Asn mutation via both Sanger sequencing and Multiplex Ligation-dependent Probe Amplification (MLPA). Notably, the external MLPA report erroneously suggested a “heterozygous deletion” at the p.Ile173Asn probe site due to probe-binding interference caused by the point mutation itself.
- Mother: Reported to have a heterozygous Exon 1–3 deletion via MLPA, while her sequence analysis (twist panel) was reported as normal.

The paradox lay in the clinical reality that a seemingly homozygous child for the p.Ile173Asn mutation was born to a mother who was completely negative for this specific point mutation on routine sequence analysis.

### 2.2 In-house molecular investigation and resolution of the discrepancy

Upon the patient conceiving (confirmed at 5–6 weeks of gestation), a comprehensive familial validation study was initiated in our molecular genetics laboratory to resolve this inheritance discrepancy before finalizing prenatal management.

Our in-house MLPA validation, performed via the SALSA MLPA P050-D1 kit, confirmed that the mother harbored only a single copy of *CYP21A2* Exons 1 and 3, along with three copies of *CYP21A1P* pseudogene probes. Due to the high sequence homology between the functional gene and the pseudogene, commercial MLPA assays lack unique probes specifically targeting Exon 2. Consequently, the single-copy findings in the flanking exons indicated a segmental gene conversion tract extending from Exon 1 through Exon 3, leaving her Exon 4 locus intact on her functional wild-type allele.

**Figure 1:**
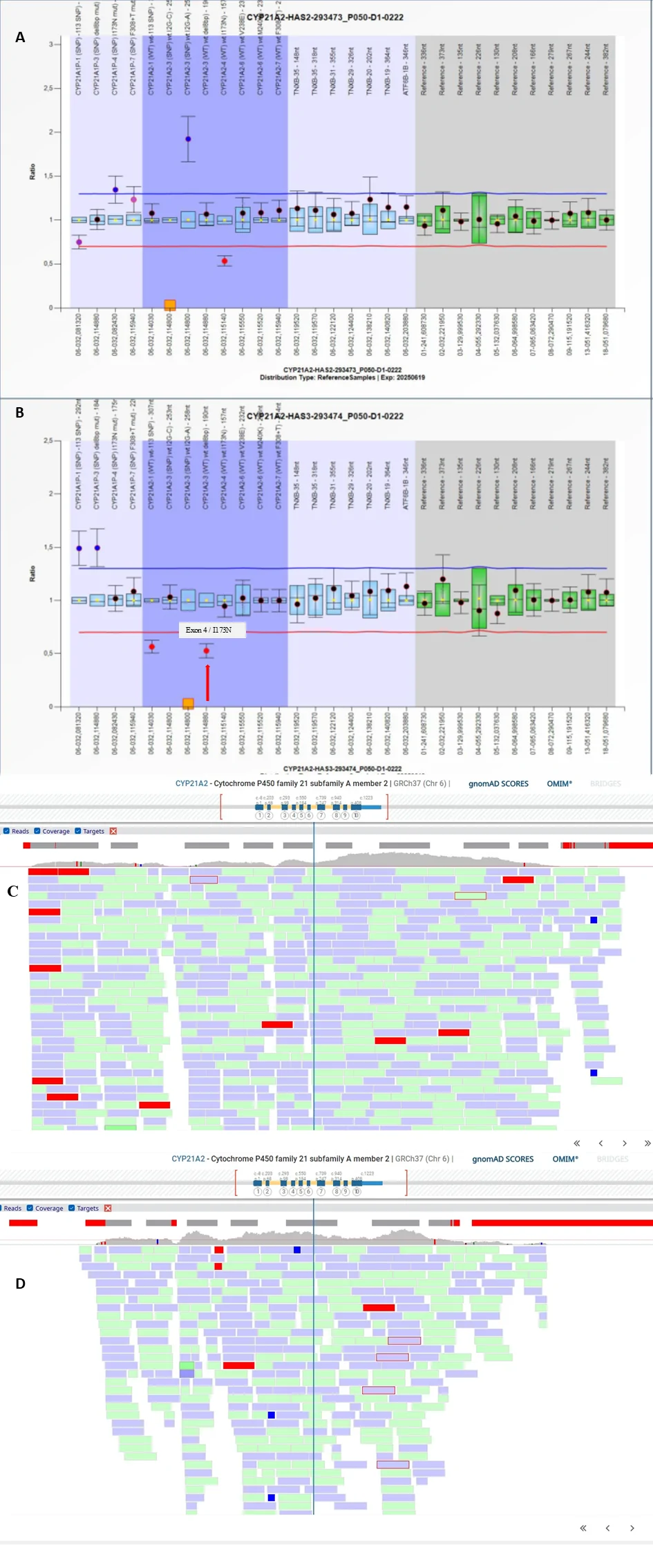
Multi-modal molecular evidence of the *CYP21A2* deletion expansion. Panels **(A, B)** show MLPA ratio charts; panels **(C, D)** show read alignment visualizations for the *CYP21A2* gene.

For the re-evaluation of the affected child (V-3), Long-Range PCR was conducted using locus-specific unique primers targeting downstream non-pseudogene regions to systematically eliminate pseudogene contamination, followed by Sanger sequencing to analyze the p.Ile173Asn region. This multi-modal approach confirmed a pseudo-homozygous state for the pseudogene-derived conversion variant, officially designated under HGVS nomenclature as NM_000500.9:c.518T>A, p.(Ile173Asn) (**Figure 1**).

Crucially, the child’s (V-3) MLPA revealed single copies of *CYP21A2* Exons 1, 3, and 4, successfully resolving the genetic paradox by demonstrating a rare intergenerational meiotic expansion of the maternal gene conversion tract. The conversion boundary shifted from Exons 1–3 in the asymptomatic mother to Exons 1–4 in her affected daughter. Because this dynamic maternal expansion physically eliminated the maternal wild-type primer binding template at Exon 4 *in cis*, standard Polymerase Chain Reaction (PCR) primers failed to amplify the normal maternal allele. Consequently, during routine sequencing, only the paternal mutant allele was captured—a classic phenomenon of allele dropout (ADO) that led to a false “true homozygous” interpretation on the external clinical report.

Biologically, this state corresponds to true functional hemizygosity *in vivo*, unmasking how a complex, dynamic structural rearrangement can mimic true homozygosity on standard clinical assays. Unmasking this pseudo-homozygosity was critically important; it allowed us to provide accurate recurrence risk assessment for the family and formulate a precise prenatal strategy for the ongoing pregnancy.

### 2.3 Re-interpretation of the father’s genetic profile

**Figure 2:**
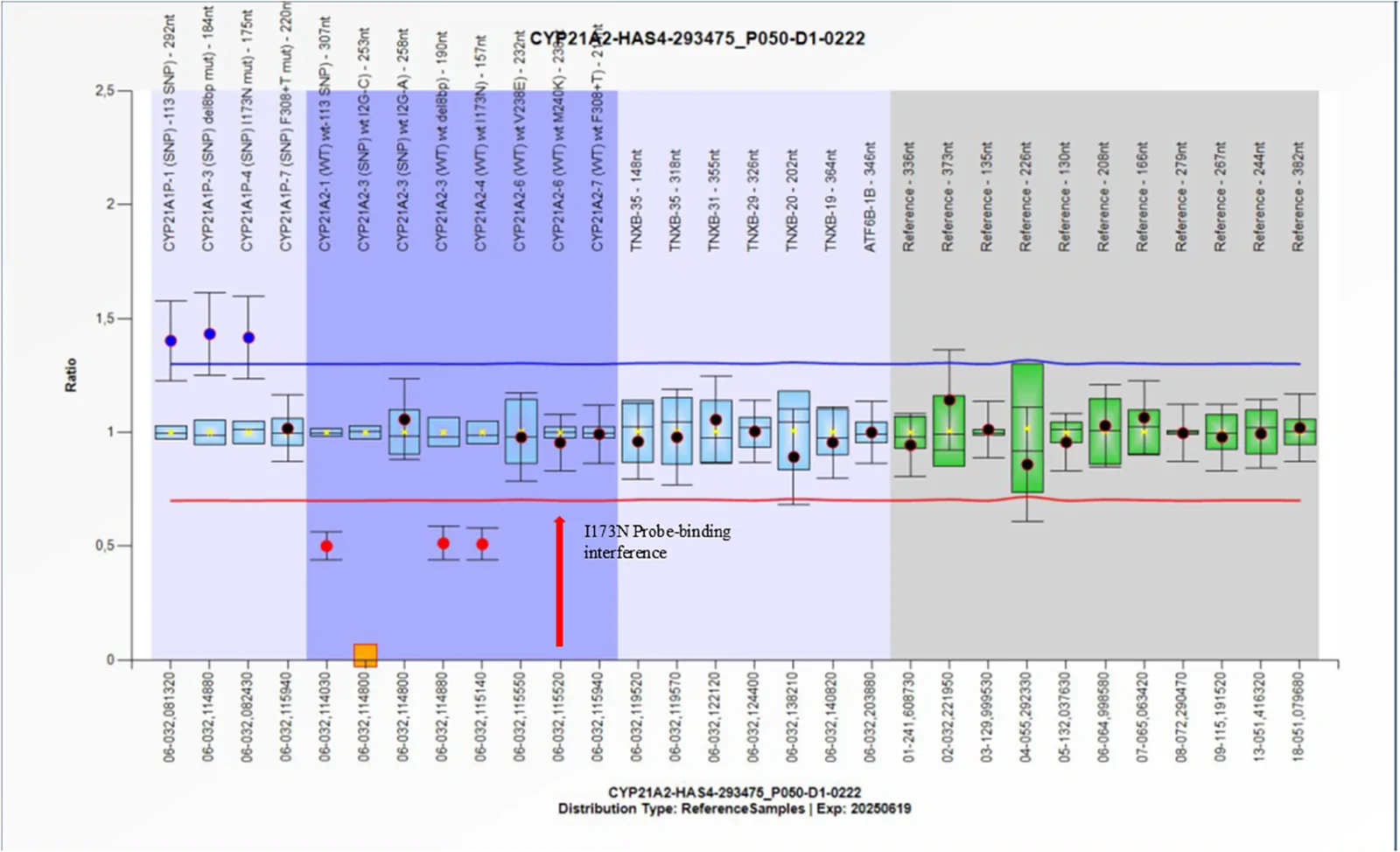
MLPA ratio chart analysis of the father (IV-1).

The “heterozygous deletion” previously reported in the father’s external MLPA analysis was systematically re-evaluated in our laboratory. This finding was determined to be a false-positive deletion signal caused by probe-binding interference rather than a true structural rearrangement. The presence of the heterozygous p.Ile173Asn mutation on his paternal allele created a nucleotide mismatch directly at the MLPA probe hybridization site, disrupting proper ligation and mimicking a copy number loss. This unmasked artifact corresponds directly to the updated footnote annotations explaining probe-binding interference in **Table 1**, **Figure 2**. By confirming the intact copy number via locus-specific independent assays, this suspected rearrangement was definitively ruled out, establishing his true status as a simple heterozygous carrier.

**Table 1:** Detailed genotype and copy number variation (CNV) analysis of the family members.

Subject	*CYP21A2* (Ex 1,3)	*CYP21A2* (Ex 4)	*CYP21A1P* copy no	Sanger sequencing	Clinical status
Mother	1 copy	2 copies	3 copies	Wild-type	Asymptomatic Carrier
Father	2 copies	2 copies*	2 copies	p.Ile173Asn (Het)	Asymptomatic Carrier
Daughter	1 copy	1 copy**	3 copies	p.Ile173Asn (Pseudo-Hom**)	Classical CAH (SW)
Fetus (CVS)	2 copies	2 copies	2 copies	Wild-type	Healthy (Carrier excluded)

The table summarizes the molecular findings obtained via Multiplex Ligation-dependent Probe Amplification (MLPA) and Sanger sequencing. Copy numbers were determined based on dosage ratios (bold values denote copy number variations indicative of specific gene conversion patterns; 0.5 for heterozygous deletion, 1.0 for normal).

The father’s apparent reduced signal at *Exon 4* in initial testing represents a false-positive deletion signal caused by probe-binding interference due to the adjacent heterozygous mutation.

The daughter’s (V-3) apparent homozygosity is biologically a hemizygous state *in vivo*, caused by a cis-acting allele dropout where the maternal dynamic meiotic expansion completely eliminated the wild-type template at the *Exon 4* primer-binding zone.

### 2.4 Prenatal management and outcome

Based on the high risk of a recurrent classical CAH presentation within this consanguineous family, maternal management was proactively optimized. Following standard early pregnancy routines, the mother was immediately initiated on standard folic acid supplementation and oral micronized progesterone (2x1) for luteal support. Concurrently, to prevent potential virilization of a female fetus *in utero*, oral dexamethasone (Dekort) was initiated at a proactive dose of 0.5 mg three times daily (3 x 0.5 mg, total 1.5 mg/day) at the 5th week of gestation. At the 12th week of gestation, transabdominal Chorionic Villus Sampling (CVS) was successfully performed.

Fetal genetic analyses from the CVS revealed a normal female karyotype (46,XX). In-house molecular evaluation, comprising comprehensive Sanger sequencing of all coding exons/exon-intron junctions and copy number assessment via MLPA of the *CYP21A2* gene, demonstrated completely normal, wild-type sequences and intact copy numbers. These findings confirmed that the fetus did not inherit either the paternally derived point mutation or the maternally expanded conversion tract. The normal fetal results confirmed that the fetus had successfully inherited the functional, wild-type paternal allele and the intact maternal allele. Consequently, dexamethasone therapy was discontinued immediately upon receiving the 12th-week molecular report to minimize unnecessary fetal glucocorticoid exposure.

The pregnancy proceeded under close perinatal surveillance. At 19.2 weeks of gestation (Last Menstrual Period: September 10), a detailed target anatomical ultrasound (TA-USG) and advanced fetal echocardiography were performed. Fetal biometry was completely consistent with the gestational age (BPD: 19 mm, FL: 18 + 4 weeks, AC: 18 + 1 weeks, HC: 18 + 2 weeks, HL: 18 + 5 weeks, and Estimated Fetal Weight [EFW]: 236 g). The placenta was located anteriorly, the amniotic fluid index (AFI) was adequate, and no retroplacental or subchorionic hematomas were detected. The cervical canal length was robust at 53 mm.

Comprehensive anatomical screening confirmed a normal fetal profile, including a well-developed nasal bone (5.4 mm, 11th percentile), clear orbital lenses, an intact palate (no cleft lip/palate), a normal intact anterior abdominal wall, normal bilateral kidneys, and a 3-vessel umbilical cord. Advanced fetal echocardiography revealed a normal left cardiac axis, a well-balanced four-chamber view with two patent atrioventricular (AV) valves, a visible moderator band in the right ventricle, normal outflow tracts (right ventricle to pulmonary artery and left ventricle to aorta), a normal great vessel crossover, a patent left atrial flap, normal superior/inferior vena cava connections to the right atrium, and an intact aortic/ductal arch with a rhythmic fetal heart rate of 149 bpm, ruling out any major cardiac anomalies. Ultrasonographic evaluation of the fetal perineum confirmed completely normal, non-virilized female external genitalia.

Although definitive chromosomal analysis options via amniocentesis and advanced non-invasive screening options (such as NIPT) were thoroughly discussed with the family at this stage, the parents formally declined further invasive diagnostic testing due to personal and ethical beliefs, opting to sign an institutional informed refusal log. The pregnancy culminated at 39.5 weeks of gestation, where a 2650-gram healthy female infant was delivered via Cesarean section (C/S) due to breech presentation. Extensive physical examination at birth confirmed a completely healthy female newborn with normal external genitalia, demonstrating the absolute clinical success of the early tailored prenatal management.

## 3 Discussion

The molecular architecture of the *CYP21A2* locus is one of the most complex in the human genome, primarily due to the tandem arrangement of the functional gene and its highly homologous pseudogene, *CYP21A1P*, within the RCCX module (1). This structural arrangement predisposes the region to frequent intergenic recombination, which can lead to dynamic changes in mutation boundaries during meiotic transmission (14, 17).

Initial external reports attributed the Mendelian discrepancy to possible technical artifacts, such as preferential amplification of the pseudogene or low-level maternal mosaicism. However, our comprehensive in-house investigation disproved these hypotheses by identifying a structural mechanism. We demonstrated that the discrepancy was not due to sequencing failure, but rather a dynamic intergenerational expansion of a maternal gene conversion tract. This rearrangement physically removed the wild-type template and caused allele dropout, a classic pitfall that standard sequencing cannot resolve on its own.

Our case highlights a remarkable and rare phenomenon: the intergenerational meiotic expansion of a maternal *CYP21A2* gene conversion, shifting from an Exon 1–3 boundary in the asymptomatic mother to an Exon 1–4 boundary in the affected offspring. The most striking finding is this *de novo* expansion, which likely occurred through a large gene conversion or an unequal crossover event during maternal oogenesis. While most *CYP21A2* rearrangements are inherited as stable segments, the repetitive nature of the RCCX region acts as a substrate for further aberrations (7, 8). Specifically, a pre-existing maternal rearrangement may serve as a ‘hotspot,’ promoting further misalignment during meiosis and leading to the recruitment of additional exonic regions into the deletion or conversion tract (7). In our case, the transition of the Exon 4 copy number from 2 (normal) in the mother to 1 (heterozygous loss) in the daughter definitively characterizes this expansion.

This structural variation rendered the paternal p.Ile173Asn allele hemizygous, leading to a pseudo-homozygous appearance in clinical sequencing reports. The tactical integration of Multiplex Ligation-dependent Probe Amplification (MLPA) and Sanger sequencing using two independent sets of unique primers verified that the daughter inherited this *de novo* expanded conversion tract from the mother and the p.Ile173Asn point mutation from the father. This established a state of compound heterozygosity that presented as pseudo-homozygosity on standard Sanger sequencing due to allele dropout of the wild-type template on the maternal allele.

This case also illustrates the critical diagnostic and clinical pitfalls associated with ‘pseudo-homozygosity.’ In the affected daughter, standard external sequencing erroneously suggested a homozygous p.Ile173Asn mutation due to allele dropout (ADO) (11, 12). Unmasking this “pseudo-homozygous” state instead of accepting a true homozygous carrier state is of monumental importance from a clinician’s and genetic counselor’s perspective (10, 13). If the index patient had been misinterpreted as truly homozygous, both parents would have been assumed to be simple, stable point-mutation carriers, completely masking the mother’s dynamic structural trap. Identifying the maternal dynamic gene conversion allowed us to:

1. Provide highly accurate recurrence risk assessments for future pregnancies in this consanguineous family (11, 18).
2. Devise a targeted, multi-modal prenatal testing strategy for the subsequent pregnancy, eliminating the diagnostic blind spots caused by allele dropout.

In clinical practice, classical salt-wasting Congenital Adrenal Hyperplasia (CAH) can cause severe virilization (Prader stages IV–V) in 46,XX female neonates, leading to erroneous male sex assignment at birth. When coupled with a false-homozygous genetic report, this can profoundly complicate and delay proper clinical management, sex reassignment, and corrective surgery, as seen in the early history of our index patient. This underscores the inherent limitations of relying solely on sequencing and highlights why dosage-sensitive assays like MLPA are mandatory for accurate genotype-phenotype correlation and correct clinical guidance (11, 18).

Furthermore, the paternal findings provided an additional layer of technical complexity. The reduced MLPA signal at the *CYP21A2* Exon 4 probe (dosage ratio: ~0.75) initially mimicked a physical structural deletion in external reports. However, we identified this as a false-positive deletion signal caused by probe-binding interference due to the adjacent heterozygous point mutation (c.518T>A) itself. This unmasked artifact corresponds directly to the updated footnote annotations explaining probe-binding interference in **Table 1** (2). This phenomenon warns clinicians that MLPA signals must be interpreted with extreme caution when sequence variants are present at the probe hybridization site, as an incorrect deletion call can severely distort pedigree analysis and genetic counseling (2).

The integration of advanced molecular strategies is increasingly recognized as the new standard for managing such complex loci. While Long-Read Sequencing (LRS) is emerging as a powerful tool to resolve difficult rearrangements and CAH-X chimeras, our case demonstrates that a well-executed combination of MLPA and Sanger sequencing using targeted unique primer sets remains highly effective and accessible for clinical management when interpreted with expert genetic counseling (9, 14, 15). A minor limitation of our study is the lack of long-read sequencing to physically map the exact breakpoints of the expanded maternal conversion tract; however, the absolute segregation of copy number variations across three generations in our family provides definitive evidence for the proposed meiotic expansion mechanism.

Finally, the successful prenatal management of the ongoing fourth pregnancy represents the optimal clinical translation of our molecular findings (5, 19). Adhering to early proactive intervention, the mother’s management was optimized with standard folic acid supplementation, micronized progesterone (2x1) for luteal support, and oral dexamethasone (Dekort) at a dose of 0.5 mg three times daily (3 x 0.5 mg, total 1.5 mg/day) initiated at the 5th week of gestation to prevent potential *in utero* virilization (5, 19). Following successful Chorionic Villus Sampling (CVS) at the 12th week, the documentation of completely normal, wild-type *CYP21A2* sequences and a normal 46,XX female karyotype allowed for the immediate and safe discontinuation of maternal steroids, successfully avoiding prolonged, empirical, and potentially harmful prenatal glucocorticoid exposure (5).

At 19.2 weeks of gestation (Last Menstrual Period: September 10), despite comprehensive perinatology counseling regarding advanced non-invasive (NIPT) and definitive invasive screening options (amniocentesis), the parents actively chose to decline further testing due to their strong psychosocial and ethical commitment to the pregnancy, signing an institutional informed refusal log. Close longitudinal follow-up, including detailed mid-trimester targeted anatomical ultrasound (confirming BPD: 19 mm, FL: 18 + 4 weeks, AC: 18 + 1 weeks, HC: 18 + 2 weeks, and EFW: 236 g) and advanced fetal echocardiography (confirming a normal cardiac axis, four-chamber view, great vessel crossover, and rhythmic rate of 149 bpm), confirmed completely normal anatomical development and ruled out any major structural or cardiac anomalies. Ultrasonographic evaluation of the fetal perineum further confirmed completely normal, non-virilized female external genitalia.

This meticulously managed strategy culminated at 39.5 weeks of gestation with the safe Cesarean delivery (C/S) of a healthy, non-virilized 2650-gram female infant due to breech presentation, demonstrating that precise molecular resolution combined with rigorous perinatological tracking is the cornerstone of successful interdisciplinary care (5, 19).

**Figure 3:**
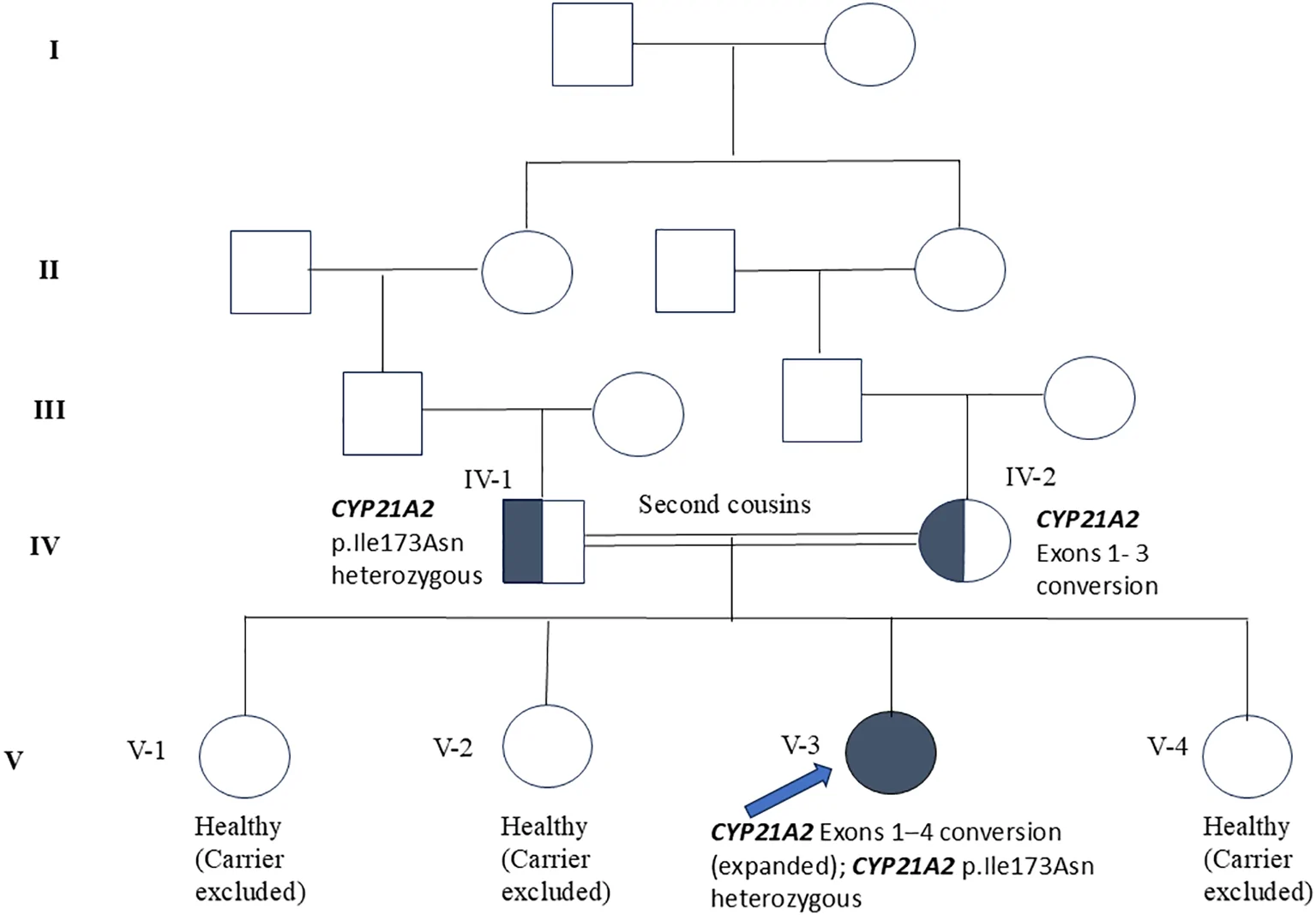
Comprehensive family pedigree and genetic segregation analysis across five generations.

The pedigree maps the inheritance pattern and dynamic meiotic rearrangement within the consanguineous family (**Figure 3**). The pedigree (illustrated in image_80ee79.png) maps the inheritance pattern and dynamic meiotic rearrangement within the consanguineous family. Roman numerals (I–V) denote generations, and Arabic numerals indicate individuals within Generation IV and V. Double horizontal lines represent consanguinity (second cousins).

- IV-1 (Father): Depicted as a heterozygous carrier for the *CYP21A2* p.Ile173Asn point mutation on his paternal allele.
- IV-2 (Mother): Asymptomatic carrier harboring a segmental maternal gene conversion tract extending from Exon 1 through Exon 3 on one allele.
- V-1, V-2, and V-4 (Healthy Offspring): Represent the three healthy daughters in whom carrier status for both the point mutation and the structural rearrangement was definitively excluded via molecular testing.
- V-3 (Index Patient): The affected third child (indicated by the solid symbol and arrow) presenting with classical salt-wasting CAH. Molecular resolution unmasked a state of compound heterozygosity presenting as “pseudo-homozygosity” due to an intergenerational meiotic expansion of the maternal gene conversion tract (shifting from an Exon 1–3 boundary in the mother to an Exon 1–4 boundary in the daughter), which triggered allele dropout of the maternal wild-type template during standard sequencing, leaving only the paternal *CYP21A2* p.Ile173Asn mutant allele detectable.

## Data Availability

The original contributions presented in the study are included in the article/supplementary material. Further inquiries can be directed to the corresponding author.
